# Lightweight Software Architecture Evaluation for Industry: A Comprehensive Review

**DOI:** 10.3390/s22031252

**Published:** 2022-02-07

**Authors:** Mahdi Sahlabadi, Ravie Chandren Muniyandi, Zarina Shukur, Faizan Qamar

**Affiliations:** Centre for Cyber Security, Faculty of Information Science and Technology (FTSM), Universiti Kebangsaan Malaysia (UKM), Bangi 43600, Selangor, Malaysia; sahlabadi2002@gmail.com (M.S.); ravie@ukm.edu.my (R.C.M.); zarinashukur@ukm.edu.my (Z.S.)

**Keywords:** software architectural evaluation, lightweight software architecture, heavyweight software architecture, software quality

## Abstract

Processes for evaluating software architecture (SA) help to investigate problems and potential risks in SA. It is derived from many studies that proposed a plethora of systematic SA evaluation methods, while industrial practitioners currently refrain from applying them since they are heavyweight. Nowadays, heterogeneous software architectures are organized based on the new infrastructure. Hardware and associated software allow different systems, such as embedded, sensor-based, modern AI, and cloud-based systems, to cooperate efficiently. It brings more complexities to SA evaluation. Alternatively, lightweight architectural evaluation methods have been proposed to satisfy the practitioner’s concerns, but practitioners still do not adopt these methods. This study employs a systematic literature review with a text analysis of SA’s definitions to propose a comparison framework for SA. It identifies lightweight features and factors to improve the architectural evaluation methods among industrial practitioners. The features are determined based on the practitioner’s concerns by analyzing the architecture’s definitions from stakeholders and reviewing architectural evaluation methods. The lightweight factors are acquired by studying the five most commonly used lightweight methods and the Architecture-based Tradeoff Analysis Method (ATAM), the most well-known heavyweight method. Subsequently, the research addresses these features and factors.

## 1. Introduction

It is essential to detect software architecture (SA) problems before software development, but it is not easy to analyze SA because of software heterogeneity and the arrangement of software components [1,2]. Consequently, the problematic SA leads to project failure; therefore, different big, upfront designs are sketched based on the experiences of software analysts to explore the SA problems in the early phase [3]. Modern SA works based on various Flask web servers and local Raspberry servers, connected via cloud technology to a central platform. They receive user requests and control the protocol through a REST API to command microcontrollers and detect sensor faults. Internet of Things (IoT) devices with resource limitations are programmed with high-complex languages, such as Python, C++, and even Java, which significantly differ from the traditional monolithic SA. It is very difficult to analyze the intercommunication of all those elements [4,5].

A successful software project delivers the agreed-upon functionalities in the software within the triangle of a specific time, budget, and acceptable quality [6]. An SA plays a vital role in this triangle since the SA initializes system design with models and analysis to ensure that the design meets the system’s functional and non-functional requirements. It extends and sustains the system by integrating it with other systems. Changes to the existing requirements always happen and may change the SA, bringing massive code rework and impacting the schedule and budget [7].

In recent years, many researchers have proposed different SA evaluation methods to uncover SA problems systematically. Generally, SA evaluations are beneficial and economical to detect the early stage’s risks or issues, but they are heavyweight and costly to maintain [8]. Despite the crucial role of architectural evaluation and many SA evaluation methods proposed by research communities, the industry only occasionally practices these methods [9]. Researchers proposed various SA evaluation methods based on different techniques such as scenario, simulation, etc. For example, the most popular scenario-based methods are different versions of the ATAM, the Software Architecture Analysis Method (SAAM), and Performance Assessment of Software Architecture (PASA). These methods, which are expensive and time-consuming, might be applied in the different software development stages. Moreover, the practitioners do not acquire business values for these assessments, while several stakeholders must participate in the SA’s documentation [10]. They are inefficient in coping with this complex technical topic since they rely on limited scenarios [11,12,13]. As a result, other approaches such as simulation and mathematics were used to promote the methods; however, the architectural evaluation methods are still heavyweight [14].

These limitations have triggered another wave of research for lightweight SA evaluation methods such as lightweight ATAM, Pattern-Based Architecture Reviews (PBAR), and Active Reviews for Intermediate Designs (ARID). It also helps to reduce the cost of the assessment process, time, and effort by minimizing the SA documentation, formality, and involved stakeholders. Although researchers took a step towards practitioners’ needs, similar to the non-lightweight methods, the lightweight methods are not being used widely in the industry [15,16].

The persistence of not using SA evaluation methods brings up the following research questions: Q1: What features and influencing factors encourage the industry to use lightweight SA evaluations? This exploratory research aims to identify the features and characteristics that enable lightweight SA evaluations in the industry. Q2: What are the differences between the aspects of SA’s from industry and academic backgrounds? This study extracts differences between the following two major SA communities: practitioners and researchers, through the comparative text analysis of SA definitions and the systematic literature review of existing methods.

Every SA evaluation method consists of the following three main parts: SA design and presentation concepts, SA evaluation procedures, and quality attributes (QA) of SA. This research is aligned to identify features and factors for a solution based on them. Besides, considering that SA is deeply concerned with industrial needs, this research makes an effort to find industrial views of SA to guide the researchers. The history of the SA standards, concepts, and definitions is discussed in the first two sections. The SA design methods section reviews and discusses the most commonly used SA design processes, styles, and presentations. In the SA evaluation section, all the SA evaluation methods are briefly reviewed and examined to identify criteria for comparing SA evaluation methods. Then, comprehensively, the most commonly used lightweight approaches are discussed to identify their strengths and weaknesses. Ultimately, this comprehensive comparison determines the lightweight factors of the SA evaluation methods. Then, there are discussions about the most concerning targeted QAs. In the end, the research discusses the identified features and factors and their relationships. Then, the next section discusses the achievements and results. Lastly, the conclusion section wraps up all the discussion. Research generally offers an added value to SA evaluation by the evaluation approach’s overview. It contrasts the benefits and drawbacks of proposed approaches and presents their constraints. Furthermore, the approaches are closely interrelated and similar. As a result, it is significant to compare existing approaches to help software architects select the most viable option from a set of approaches.

## 2. Software Architecture Definition Differences

Although SA has been used for decades, professionals still cannot refer to a single SA definition as stakeholders recognize “architecture” based on their interests [17,18,19,20]. Therefore, it leads to different purposes for SA, where standards derive between common vocabularies and understanding. The standard IEEE 1471, and its refined successor version ISO/IEC/IEEE 42010: 2011, tried to address stakeholders’ concerns, but researchers and specialists did not give major attention to these standards as they were general [21]. The SA standards are naturally genuine standards following a “late adaption,” as they emerged because of awareness of their usage. They are also an “open standard” that permits more people to get involved in the standard’s definition.

Moreover, this standard may apply the same methods to a wide range of conditions, so the stakeholders avoid SA standards [22]. As SA standards are a type of late adaption and SA was established in the industry, academics follow it. Therefore, to challenge this academic research, when the new SA trends originating from the industry are recognized, this academic research can be associated with these trends. Therefore, the parties to the standards are the academic and practitioner architect communities. This research seeks the differences between academic and industry definitions of SA by applying the text-analyzing technique to a web definition repository by using data mining, which identifies new SA trends.

The Software Engineering Institute of Carnegie Mellon University provides comprehensive online repositories of SA definitions [23]. Retrieved data of community SA definitions are compared with classical, bibliographical, and modern SA descriptions. These definitions are divided into the following two groups: First, community SA, which collects practitioners’ definitions, and second, academic and conventional definitions. First, the keywords from the two groups of definitions are extracted, and then the buzzwords are omitted. Second, the words are rooted, and some terms that refer to the same concept are considered one word concerning the three elements of SA definitions (component, connector, and constraint) [24]. For instance, (connector, glue), (component, service, element, unit, module), (*constraint*, limitation), etc. In the end, 127 words are recognized for each group, and the words are ranked based on their occurrences. The most frequent keyword between communities is “design”. Differences between the existing definitions illustrate the real need for SA, which practical stakeholders have perceived. The result indicates “Time”, “Cost”, “Complexity”, and ‘Distributed’ are widespread words that belong to community SA definitions that are not in academic definitions of keyword lists.

Meanwhile, for the academic group’s sake, this study refers to Muccini et al. [25], which conducted a systematic review by data mining techniques on the selected topics in SA, containing 811 published studies from 1999 to 2016. The result indicates most of the studies are concerned about “performance and security analysis”. Moreover, there are new rising trends in SA’s agility that are mostly distributed and heterogeneous systems, which have been investigated as an application area due to their complexity.

The default antonym of “Time”, “Cost”, and “Complexity” means ***lightweight***, which is fast, cheap, and straightforward [26]. “Distributed” systems are “heterogeneous” and intricate systems that derive from complex SA [27]. It means that “Complexity” increases the “Cost” and “Time” of the SA design and evaluation. Therefore, this research implies that SA communities need a lightweight SA evaluation framework, which analyzes distributed and the performance and security of heterogeneous systems.

It refers to overall performance and security, two qualities concerned with the system execution. Security may vary radically from one case to another, but performance is broadly the system’s responsiveness and time of interaction between software components. These qualities are discussed in more detail in Section 5.1 and Section 5.2.

## 3. Software Architecture Design

The lack of standards in SA presentations may cause a mismatch between the SA presented and the actual SA. The Unified Modeling Language (UML) has become an international standard, ISO/IEC 19505: 2012, and has been accepted as an effective industrial standard for SA [28]. However, UML notation meets the user’s needs and is flexible enough to follow their expectations; this flexibility is embedded in semantic informality that can be understood differently [29,30]. Some researchers [27,28,29,30,31,32,33] imply that UML per se is not enough, and there is a need for a formal approach to adapt the UML. Moreover, Rodriguez et al. [31] and Medvidovic et al. [32] proved Petri net supremacy over architectural description languages (ADLs) and formal methods languages. As a result, Petri net can bridge this gap. Additionally, Jensen et al. [33] and Emadi et al. [34] stated that timed hierarchical colored Petri net is a compatible version of Petri net that can be utilized to simulate complex data values and SAs [35].

Architectural patterns are an ideal complement to architectural decisions. An architectural design, interchangeably called architectural style, is specified as a set of principles besides a coarse-grained pattern abstract framework for systems [36,37]. It is a standard solution, reused and partitioned for chronic issues in the SA area. An architectural style thoroughly regulates the vocabulary of components and connectors. It means how they can be together with a set of constraints. It may impose some topologic restraints on architectural explanations [38].

Additionally, it may have some execution semantics, which can be part of the style definition [39,40]. For the rising concern of distributed and heterogeneous software mentioned in the previous section, the commonly used architectural styles are client/server, component-based architecture, domain-driven design, layered architecture, message bus, N-tier/3-tier, object-oriented, SOA, and pipe and filter. In practice, the standard SA of a system is mainly made up of a pattern of different architectural styles for systems [41].

Despite the popularity of domain-driven design, layered architecture, message bus, N-tier/3-tier, and object-oriented modeling, it is challenging to scale them up. Moreover, there is no tool to analyze or measure non-functional properties. The interconnection mechanisms are so basic (method invocation); thus, complex interconnections are so hard in these methods. In a nutshell, there is no clear image of system architecture before component creation [42]. Component-based SA relieves this modeling trouble and develops reusable off-the-shelf component-based heterogeneous systems.

In comparison with other SA styles, a component or connector has a higher level of roughness. ISO/IEC/IEEE 42010: 2011 defines this architectural type as an effective standard [43]. Along with component-based architecture, SOA, and pipe and filter facilitate the handling of non-functional properties. It has the excellent capability of making complex products, apart from what kind of platform or technology is used in these products. However, in comparison, the presentation is still a problem in both styles [44,45,46,47].

## 4. Software Architecture Evaluation

This section examines the existing literature on SA evaluation for the last three decades to answer research questions about SA evaluation [48]. This systematic review results in the factors that can be used for proposing an evaluation framework.

After more than 30 years of SA evaluations, many research questions remain open about the categorization of SA evaluations. This section determines the criteria for the classification of SA evaluations to identify factors for SA evaluation. In this study, for the systematic review of the literature, the terms of SA “Review”, “Evaluation”, “Analysis”, “Assessment”, and “Validation” are used interchangeably as search keywords.

**Figure 1:**
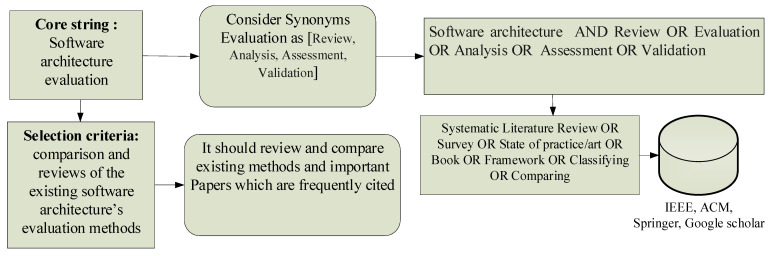
The strategy for selecting credential research and SA evaluation methods.

Figure 1 and Table 1 indicate that 27 credential SA evaluations have been selected to review from IEEE, Springer, ACM, Elsevier, and Google Scholar. They are the standard methods and techniques of SA evaluation.

Although SA evaluation is an important activity at any stage of the software life cycle, it is not widely practiced in the industry [64]. Gorton et al. [65] conducted large-scale research to identify the industrial practices of architecture evaluations to categorize SA evaluations based on evaluation techniques. Table 2 indicates the investigations in architectural evaluations from the industrial aspect. The techniques and methods listed in the first column of Table 2 are based on the frequency of use in industry; these techniques are used in the evaluation methods. The most frequent methods of each technique are listed below, and the quality attributes (QAs) are applied to them. These methods are elicited from systematic literature reviews and the latest related academic papers and books.

Despite the encouraging number of basic research found (76 approaches), it is evident that only 27-SA evaluation approaches, regardless of their targeted QAs. This table tries to compare the techniques of approaches. Later on, in Section 5, the target quality attributes will be discussed. There are some reasons for such a massive decrease in the amount of research. First, there were some identical entries for the same article when we searched in numerous databases. Second, a large percentage of the study evaluated one or several QAs in a subtle ad hoc way. Consequently, those studies are omitted, as they did not manuscript a repeatable evaluation process or method. Third, some studies considered both software and hardware evaluations, so they were not suitable in the current research emphasizing SA evaluation approaches. Below, the techniques and methods are discussed.

- The first technique focuses on experience, where SA plays a vital role in design and evaluation [66]. This is the most practiced technique by the industrial section [56]. Empirically Based Architecture Evaluation (EBAE) is performed late in development. At the same time, Attribute-Based Architectural Styles (ABAS) can run during the design time and get integrated with ATAM [49,60,67]. Decision-Centric Architecture Reviews (DCAR) analyse a set of architectural decisions to identify if the decision taken is valid. It is more suitable for agile projects due to its lightweight [62].
- The second-most popular technique is the prototype that collects early feedback from the stakeholders based on and enables architecture analysis to look at close-to-real conditions. It may answer questions that cannot be resolved by other approaches [68].
- The third technique is a scenario-based evaluation. SAAM is the earliest method using scenarios and multiple SA candidates. Later on, ATAM completed SAAM by trade-off analysis between QAs, where ATAM uses qualitative and quantitative techniques. The Architecture-Level Modifiability Analysis (ALMA) and Performance Assessment of Software Architecture (PASA) have been used to combine scenarios and quantitative methods to boost the results [69,70].
- The fourth technique is checklists, consisting of detailed questions assessing the various requirements of architecture. Software Review Architecture (SAR) uses checklists according to the stakeholder’s criteria and the system’s characteristics. The Framework of Evaluation of Reference Architectures (FERA) exploits the opinions of experts in SA and reference architectures. There is a need for a precise understanding of the requirements to create the checklist [71,72].
- The fifth technique is simulation-based methods, which are very tool-dependent; the Architecture Recovery, Change, and Decay Evaluator/Reference Architecture Representation Environment (ARCADE/RARE) simulates and evaluates architecture by automatic simulation and interpretation of SA [73]. An architecture description is created using the subset of toolset called Software Engineering Process Activities (SEPA), descriptions of usage scenarios are input to the ARCADE tool [74]. Many tools and toolkits transform architecture into layered queuing networks (LQN) [75]. It requires special knowledge about the component’s interaction and behavioral information, execution times, and resource requirements [76]. Formal Systematic Software Architecture Specification and Analysis Methodology (SAM) follows formal methods and supports an executable SA specification using time Petri nets and temporal logic [77]. It facilitates scalable SA specification by hierarchical architectural decomposition.
- The sixth category is for metrics-based techniques that need to be mixed with other techniques, and they are not intrinsically powerful enough [78]. Here are some examples of metric-based methods. The Software Architecture Evaluation on Model (SAEM) is based on the Goal/Question/Metric Paradigm (GQM) to organize the metrics. Metrics of Software Architecture Changes based on Structural Metrics (SACMM) measures the distances between SAs endpoints by graph kernel functions [79]. Lindvall et al. [60] introduced late SA metrics-based approaches to compare the actual SA with the planned architecture.
- The seventh technique, focusing on mathematical-model-based methods, is highlighted in the research areas, but the industry does not attend to them. Software Performance Engineering (SPE) and path and state-based methods are used to increase the reliability performance. These modeling methods exploit mathematical equations resulting in architectural statistics such as the mean execution time of a component and can be mixed with simulation [80,81].

Concisely, all the above discussion derives is that scenarios-based evaluation techniques are well-investigated and broadly reviewed in research papers. It has been completed by other techniques, such as simulations and mathematical techniques, to perform effectively. Another technique is simulation, focusing on the main components of a planned or implemented architecture to simulate context. While mathematical techniques use static evaluation of architectural designs, some modeling techniques originate from high-performance computing and real-time systems. Experienced-based techniques are different from other techniques. They are less explicit, and they are based on subjective factors, namely, intuition and experience. This technique is based on reviewer perception, objective argumentation, and logical reasoning. For example, an expert might recognize an availability problem, and then they convince others through scenarios that depict the situation.

**Table 2:** SA evaluation categorization.

Techniques	Methods	Quality Attribute	Remarks
Experience-based Experts encountered the software system’s requirements and domain.	EBAE (Empirically Based Architecture Evaluation)	Maintainability	The most common technique applied to review architecture in the industry [5], based on expert’s knowledge and documents.
	ABAS (Attribute-Based Architectural Styles)	Specific QAs	
	DCAR (Decision-Centric Architecture Reviews)	All	
Prototyping-based Incrementally prototyping before developing a product to get to know the problem better.	Exploratory, Experimental, and Evolutionary	Performance and modifiability	The delighted techniques have been applied to review industry architecture; possibly an evolutionary prototype can be developed into a final product.
Scenario-based The specific quality attribute is evaluated by creating a scenario profile conducting a concrete description of the quality requirement.	SAAM (Software Architecture Analysis Method)	All	Scenario is a short description of stakeholders’ interaction with a system. Scenario-based methods are widely used and well known [49].
	ATAM (Architecture Trade-off Analysis Method)		
	Lightweight-ATAM (derived from ATAM)		
	ARID (Active Reviews for Intermediate Designs)		
	PBAR (Pattern-Based Architecture Reviews)		
	ALMA (Architecture Level Modifiability Analysis)	Modifiability	
	PASA (Performance Assessment of Software Architecture)	Performance	
Checklist-based A checklist includes a list of detailed questions to evaluate an architecture.	Software Review Architecture (SAR)	All	It depends on the domain, meaning that a new checklist must be created for each new evaluation.
	FERA (Framework of Evaluation of Reference Architectures)		
Simulation-based It provides answers to specific questions, such as the system’s behavior under load.	ARCADE/RARE (Architecture Recovery, Change, and Decay Evaluator/Reference Architecture Representation Environment)	Performance	There is a tendency to mix simulation and mathematical modeling to extend the evaluation framework.
	Layered Queuing Networks)		
	SAM (Formal Systematic Software Architecture Specification and Analysis Methodology)		
Metrics-based It uses quality metrics to evaluate architecture and its representation.	SACMM (Metrics of Software Architecture Changes based on Structural Metrics)	Modifiability	It is not rampant in the industry. Commonly other methods exploit metrics to boost their functionalities.
	SAEM (Software Architecture Evaluation on Model)		
	Design pattern, conformance with design and violations.	Inter-module coupling violation.	
	TARA (Tiny Architectural Review Approach)	Functional and non-functional	
Math Model-based By mathematical proofs and method, operational qualities requirements such as performance and reliability are evaluated.	Path and state-based methods	Reliability	It is mixed with a scenario and simulation-based architecture to have more accurate results.
	SPE (Software Performance Engineering).	Performance	

### 4.1. Categorizing of Software Architecture Evaluation

There is no distinctive categorization in technique-based classifications because hybrid architecture evaluation methods that use multiple architecture evaluation techniques belong to different categories [82]. The selected methods’ discussions discover various classifications, comparing them with some commonalities regarding the assessment procedure’s activities and artifacts. However, since it is not apparent which methods are the same as the proposed solution, these methods will be analyzed to attain their common aims and an objective mechanism. To address this problem, we have recognized a set of criteria that can provide a foundation for comparing and assessing SA evaluation methods.

This study proposes a comparison framework to present and compare the analysis methods to elaborate on these fundamental criteria. It has been proposed by the combination of three software evaluation comparison frameworks [83,84]. In Table 3, this comparison framework is introduced, which contains the following main components of SA evaluation methods: context, stakeholder, contents, time, and reliability. For each component, the related elements are identified and mentioned. Then the existing taxonomies of these elements are generally mentioned in taxonomic comparison, and the taxonomies are broken down in more detail in the complementary table.

Niemela et al. [85] introduced a framework to compare SA evaluation methods with some essential criteria. These criteria are listed below from C1 to C7, which can be answered concerning Table 3:

C1: The main goal of the method.
C2: The evaluation technique(s).
C3: Covered QAs.
C4: Stakeholder’s engagement.
C5: How applied techniques are arranged and are performed to achieve the method goal.
C6: How user experience interferes with the method
C7: Method validation

Other than these criteria, *tools*, and *techniques*, and *the SA description and outcomes* of the methods are also explored. Moreover, C4, C5, and C6 are replied during the review of the SA evaluation “*process*”. These criteria are used to compare the existing solution to identify factors that increase the evaluation framework’s use.

### 4.2. Identifying Factors for Lightweight Evaluation Method

Architecture evaluations are usually performed manually based on informal/semiformal architecture documentation and the reviewer’s knowledge [86]. The comprehensive SA evaluation methods, in particular ATAM, require a massive amount of cost and effort. This problem results in lightweight SA evaluation methods. Heavyweight reviews are long-running, documentation-based, such as technical reviews and inspections. While lightweight runs are based on short-running processes with little architecture documentation. The criteria for lightweights have not yet been defined, and they are detectable based on the publication’s claim. In this research, ATAM, as the best sample of heavyweight methods, ARIS, BAR, and TARA,—ATAM, ARID, PBAR, and TARA, are lightweight to identify factors for lightweight methods [87,88].

#### 4.2.1. Architecture Tradeoff Analysis Method

ATAM is the most mature, sophisticated, and well-known method that various researchers have devised. ATAM-based methods are flexibly used for evaluation purposes, such as the following: seeking SA improvement opportunities, risk analysis, SA comparison, but generally, finding out whether the candidate SA supports business goals adequately. ATAM-based methods engage various stakeholders with various techniques for prioritizing requirements by cumulative voting and utility tree, then identify trade-offs to resolve conflicts.

C1 (Main Goal): identifying SA patterns and tactics suit business derives.

C3 (Covered QAs): All QAs or any property that can affect the business goals.

C4, C5, and C6 (Process): Table 4 explains the ATAM process.

C2 (Evaluation Techniques): Based on scenario and experience.

C7 (Validation): It has been extensively validated.

Outcomes: A list of risks, non-risks, risk-themes, sensitivity points, and trade-off points.

SA description: SA styles and tactics, as well as ATAM-based methods, define a precise template for documenting the quality scenarios.

Tools and techniques: Brainstorming and voting.

Discussion: Although it exploits a scenario-based paradigm, it engages various stakeholders for up to six weeks and is costly. The quality scenarios and ATAM templates represent the requirements in detail, but they are often confusing. Cumulative voting can induce stakeholders into excessive rivalry. Moreover, its reliance on SA documentation and the ignorance of project management paradigms makes ATAM impossible to run for agile projects.

**Table 4:** The steps of ATAM.

ATAM Step (C5)	Stakeholders Engagement (C4)	How User Experience Interferes with the Method (C6)	Outputs
**First Phase: Presentation**			
1. Briefing of the ATAM	Evaluation Team and All stakeholders	The stakeholders will understand the ATAM process and related techniques.	–
2. Introduction of the business drivers		The stakeholders will understand the goals of businesses and the architectural drivers (non-functional qualities affect SA).	–
3. Introduction of SAs	Evaluation Team and who make significant project decisions	The evaluation team will review the targeted SA.	–
4. Identifying the architectural approaches	Evaluation Team and software architects	The team and architects will highlight architectural patterns, tactics, and SA design.	List of the candidate SAs design
5. Producing of the quality attribute tree	Evaluation Team and those who make significant project decisions	The decision-makers will prioritize their decision based on the quality attribute goals.	The first version is based on prioritized quality scenarios and a quality attribute tree.
6. Analyze the architectural approaches	Evaluation Team and software architects	They will link the SA to primary quality attribute goals to develop an initial analysis resulting in non-risks, risk, and sensitivity/trade-off points.	The first version of non-risks, risks, risk themes, trade-off points, sensitivity points
**Second Phase: Testing**			
7. Brainstorming and prioritizing scenarios	Evaluation Team and All stakeholders	It will utilize the involved stakeholder’s knowledge to expand the quality requirements.	The last version of prioritized quality scenarios
8. Analyze the architectural approaches	Evaluation Team and software architects	Revised the achievements.	The version of the of non-risks, risks, risk themes, trade-off points, sensitivity points
**Third Phase: Reporting**			
9. Conclusion	All stakeholders	It summarizes the evaluation’s achievements regarding the business drivers introduced in the second step.	Evaluation report with final results

#### 4.2.2. Lightweight ATAM

Costs of the ATAM-based method derived from Lightweight ATAM requires less than 6 h running. The technique is used by a development team that is familiar with ATAM, SA, and goals.

C4, C5, and C6 (Process): The evaluation process was created by eliminating or constraining the scope of ATAM’s activities, which is shown in Table 4. It assumes the participants are familiar with ATAM while brainstorming and prioritizing are omitted because of their cost.

Steps 1, 7, and 8 are omitted, and steps 2, 3, 4, 5, and 6 should be completed in 15 min. Step 9 should be completed in 30 min.

C7 (Validation): No validation, and the method features generally are as same as ATAM.

Discussion: The method reduces the stakeholder’s engagement, and the evaluation process steps, but architecture evaluation still needs more formality. It relies on the stakeholder’s familiarity and tactical knowledge, which is achieved because of the full ATAM implementation. It is evident that by constraining the scope and depth of evaluation, a lower effort is needed.

#### 4.2.3. ARID

C1 (Main Goal): Detecting of SAs issues to assess the appropriateness of the chosen SA.

C3 (Covered QAs): Applicable for any QA originated from the quality scenarios.

C4, C5, and C6 (Process):

Phase 1: Meeting for preparation.
  Step 1: Appointing of reviewers.
  Step 2: Presenting of SA’s designs.
  Step 3: Prepare seed scenarios.
  Step 4: Arranging the meeting.
Phase 2: Review meeting.
  Step 5: Presenting ARID.
  Step 6: Presenting designed SA.
  Step 7: Brainstorming and prioritizing the scenarios.
  Step 8: Conducting of SA evaluation.
  Step 9: Results.

C7 (Validation): One pilot experience in the industry.

C2 (Evaluation Techniques): Based on scenario and expertise.

SA description: There is no specific form of SA designs or documents.

Tools and techniques: Brainstorming and voting.

Outcomes: List of the given SA issues.

Discussion: It is a simple method to seek flaws and weaknesses in QAs of the given SA. ARID does not explicitly state the QAs and SA styles during the analysis. The analysis focuses on a set of properties represented by a group of quality scenarios. It has nine steps, which are not compatible with the lightweight concept. It emphasizes an expert informal review with no particular form of SA style. As a result, it is difficult to repeat.

#### 4.2.4. PBAR

ATAM is the most mature, sophisticated, and well-known method that various researchers have devised. ATAM-based methods are flexibly used for evaluation purposes, such as the following: seeking SA improvement opportunities, risk analysis, SA comparison, but generally, finding out whether the candidate SA supports business goals adequately. ATAM-based methods engage various stakeholders with various techniques for prioritizing requirements by cumulative voting and utility tree, then identifying trade-offs to resolve conflicts.

C1 (Main Goal): Detecting quality attribute issues.

C3 (Covered QAs): Potential risks influencing QAs.

C4, C5, and C6 (Process):

1. Elicitation of essential quality requirements from user stories with the assistance of developers.
2. Establishing SA’s structure by a discussion with developers.
3. Nominating architectural styles.
4. Analyzing the nominated architectural effects on the qualities.
5. Recognizing and discussing the final results.

C2 (Evaluation Techniques): It is based on scenario and experience.

C7 (Validation): Nine student small-size projects for industrial use.

SA description: There is no specific form of SA designs or documents, but SA styles are included during the evaluation.

Tool and techniques: Informally requirement elicitation during the development team meeting.

Outcomes: It has the QAs issues, which are mismatches between QAs and SA styles.

Discussion: PBAR contains all the criteria of the lightweights. It reduces the process into five steps that occur once in face-to-face meetings with the development team. It omits the prioritizing requirements to help the method. PBAR requires a negligible amount of time to run in comparison with the traditional methods. It focuses on the production step in agile projects. It is operational in the software industry rather than the conventional methods for companies that use agile and lean software development methodologies. It also confines the use of this method comprehensively. The evaluation uses SA styles and tries to find mismatches between SA styles and QAs of candidate SAs. However, it ignores formalizing the assessment technique and merely relies on tacit knowledge of SA styles and their impacts on QAs. Moreover, the influence of styles on QAs is not conclusive in most cases since other factors should be taken into account.

#### 4.2.5. TARA

C1 (Main Goal): Indicating the proper SA of crucial requirements.

C3 (Covered QAs): QAs and even functional requirements.

C4, C5, and C6 (Process):

1. The evaluator elicits essential requirements and system context.
2. The evaluator designed SA based on the previous.
3. The implementation techniques are assessed.
4. The results of the previous step should link to the requirements. Expert judgment techniques are applied in this step.
5. The evaluation’s results should be collected and related based on the predefined forms.
6. Present findings and recommendations.

C2 (Evaluation Techniques): Metric-based.

C7 (Validation): TARA has been validated in the industry.

SA description: There is no specific form of SA designs or documents, but the evaluator should understand functional/deployment structures and system context.

Tool and techniques: The method involves automated code analysis techniques (module dependencies, size measures, code metrics, and test coverage). For implemented software exploits information on software execution (e.g., event logs).

Outcome: a list of crucial requirements with its relevant SA.

Discussion: TARA is a lightweight permissive method that does not exclude requirements specification documents. It allows an evaluator to consult with the stakeholders to prioritize the requirements. TARA suits the implemented software since it uses code analysis techniques with operational data. Evaluation methods mainly rely on explicit scenarios and the architect’s knowledge, but TARA relies on the reviewer’s judgment associated with the SA analysis evidence. Consequently, it just works well for implemented software in the maintenance phase when it is hard to correct the flaws.

#### 4.2.6. DCAR

C1 (Main Goal): Suitability of architectural decisions.

C3 (Covered QAs): A set of architectural decisions.

C4, C5, and C6 (Process):

1. Preparation: The SA styles and related technologies are presented for management and customer representatives.
2. DCAR Introduction.
3. Management presentation: The management/customer representative will be exposed to a brief presentation to elicit the potential decision forces (the list of architectural decisions was produced in the first step).
4. Architecture presentation: The lead architect will present potential decision forces and potential design decisions to all participants in a very brief and interactive session to revise the list of architectural choices.
5. Forces and decision completion: The decision forces and design decisions will be verified based on the same terminologies for all stakeholders.
6. Decision prioritization: The decisions will be prioritized based on participant’s votes.
7. Decision documentation: The most important decisions will be documented in applied architectural solutions, the addressed problem, the alternative solutions, and the forces that must be considered to evaluate the decision.
8. Decision evaluation: By discussion among all stakeholders, the potential risks and issues are selected, the decisions are revised based on decision approval.
9. Retrospective and reporting: Review team will scrutinize all the artifacts and produce the final report.

C3 (Evaluation technique): Experience-based and expert reasoning.

C7 (Validation): It has been verified in five large industrial projects.

SA Description: SA design, informal requirements, and business drivers.

Output: Issues and risks.

Tools and technique: Templates, wiki, and UML tools.

Discussion: DCAR originated from SA evaluation experiences in the industry. It is a lightweight method that allows users to analyze and record the rationale behind architectural decisions systematically. In comparison, scenario-based methods test SAs against scenarios to find flaws and issues in a specific QA. For the sake of being lightweight, brainstorming and prioritizing steps are omitted. The reviewers should know SA and rely on the standard UML tool to make the evaluation understandable for stakeholders. Although it provides comprehensive templates for assessment, it considers several factors that originated from managerial views. This consideration leads to the nine steps, which are not compatible with lightweights.

### 4.3. Factors for Lightweight Evaluation Method

The five lightweight methods plus ATAM are compared in Table 5 based on the following most common aspects: the evaluation methods, SA description, evaluation time, method’s validation, and tool support. These aspects are categorized based on the comparison framework reflected in Table 3, and the approaches are related to them.

#### 4.3.1. Covering Early and Late Methods

SA has been evaluated at various points in the software life cycle. It can happen at the early and late stages of the development life cycle. Early methods evaluate SA candidates before the implementation, while late methods assess the system’s implemented versions compared to the planned/previous versions. Early methods are based on SA descriptions and other sources of information. These methods lead to a better understanding of SA and the identification of problems with the architecture. At the same time, late processes utilize data obtained from the actual software implementation. Hence, the existing architecture can be reconstructed to compare with early evaluated SA. Early methods mostly contain scenario-based, mathematical-model-based, and simulation-based, while late ones are mostly metrics-based and tool-based. Early methods emphasize designing and modeling while late ones try to catch code violations and module inconsistencies. Sometimes, early methods can evaluate the implemented software [89,90]. While late and early evaluation is not contradictory, they can mostly not be attended simultaneously due to the overload they imbue on the approach. As it is indicated in the time of the evaluation part of Table 5, no method can cover all the stages.

#### 4.3.2. Need of Agility

Although SA evaluation is beneficial, it is not broadly applied in the industry nowadays. Even agile development approaches do not encourage using architecture evaluation methods since they usually take a considerable amount of time and resources [91,92]. Except for PBAR and DCAR, the other methods are not proper for agile projects.

#### 4.3.3. Ad Hoc Analysis

Ad hoc analysis ties architecture analysis to architecture design and implementation activities employing experience, expertise, and argumentation [93]. Informal experience-based architecture analysis is prevalent, as this method works regardless of architecture documentation. This analysis is carried out manually by several SA studies [94,95].

## 5. Targeted Quality Attributes: Performance and Security in Software Architecture Evaluation

Based on “software architecture definition differences” in this research, performance and security are selected as the targeted QAs. These QAs will be discussed in the following subsections. The QAs belong to the methods represented in Table 2.

### 5.1. Performance

**Figure 2:**
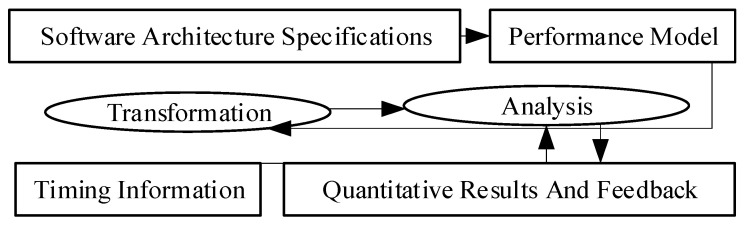
Software architecture-based performance analysis.

Mostly, the performance is estimated based on the approximate model of the runtime view. These methods need appropriate descriptions of the dynamic behaviors of SA to show the characteristics of the components, frequency, and nature of inter-component communication. Mathematical formalism such as Petri net and simulation boost this estimation [96]. Figure 2 [48] shows that most SA performance analysis methods convert SA specifications to desirable models. Subsequently, timing data is added to the models to estimate performance attributes and provide the following feedback:

- Predicting the system’s performance in the early stages of the software life cycle.
- Testing performance goals.
- Comparing the performance of architectural designs.
- Finding bottleneck, possible timing problems.

Some of the essential methods are discussed in the following: CF is a mathematical model-based method that integrates performance analysis into the software development cycle. It presents software execution behaviors through a graph, including arcs and nodes, with timing information. This model works based on the Queuing Networks Model (QNM) performance model. The simulation evaluates the model to estimate performance attributes. The approach has been enriched by using Kruchten’s 4 + 1 views and using use case scenarios depicted by a message sequence chart as the dynamic behavior of SA. Later on, the approaches combine UML diagram information to create a performance model of SA more formally. However, the methods do not consider the concurrent/non-deterministic behaviors of the components during QNM modeling. In order to address these problems, the labeled transition system (LTS) graph and ADL were added to the approaches [97,98]. The emerging problem was the computational complexity of the possible state space explosion of the architecture description’s finite-state model. This problem persuades experienced-based analysis methods such as ABAS to not use an analysis tool to evaluate performance [99].

PASA boosts SPE by adding performance anti-patterns and architectural styles. It tries to adapt the concept of ATAM and SAAM into SPE. PASA formally states the scenarios with a descriptive architecture language such as the UML sequence diagram [100].

Nevertheless, none of these approaches has yet been applied to a complete environment for performance analysis, specification, and providing feedback to the designer. The unsolved problem is automating completely derived performance models from the software specification and assimilating the supporting tools in a comprehensive environment [101]. Moreover, although the quality of models has not yet been attended to deeply, high-quality models are an essential factor in which verification and performance analysis strongly rely on them [102].

### 5.2. Security

Security is a complex technical topic that can only be treated superficially at architectural levels. Although scenario-based methods are typically used for SA security analysis, security differs from other quality attributes. The security requirements are not enough for constructing a “security scenario” by themselves [103]. At the same time, it is necessary to understand the precise security requirements of an application and devise mechanisms to support SA security. In the implementation layer of SA, there are many techniques such as Windows operational security, *Java Authentication and Authorization Service* *(JAAS)*, and without any significant problems [104]. These techniques mitigate the principal threats: authorization violation, system penetration, integrity compromise, confidentiality disclosure, repudiation, and denial of service.

The most important problem is that distributed SA has multiple layers of abstraction. Once each service abstracts the lower layer’s business functionality, it is needed to abstract the underlying application’s user identity context. Combining with the individual backends, heterogeneous security concepts beget a long way from the first request for a business procedure to the systems. Therefore, it also comprises monitoring, logging, and tracing all data flows related to security [105]. Security architectural flaws can be omissions, commissions, and realization flaws [106].

- Omission flaws are born in the aftermath of decisions that have never been made (e.g., ignoring a security requirement or potential threats). Experience and prototype-based or even scenario-based methods can help the architect to detect this type of flaw. Still, they are mainly concerned with the requirement elicitation step, which is outside the scope of this research.
- Commission flaws refer to the design decisions that were made and could lead to undesirable consequences. An example of such flaws is “using a weak cryptography for passwords” to achieve better performance while maintaining data confidentiality. DCAR is devised to support such a problem.
- Realization flaws are the correct design decisions (i.e., they satisfy the software’s security requirements), but their implementation suffers from coding mistakes. It can lead to many consequences, such as crashes or bypass mechanisms. TARA and SA evaluation methods can mitigate these problems.

In the industry, commission and omission flaws happen due to inexperienced decisions. The realization flaws are mostly ignored due to the cost of the detective methods. As a result, this current research highlights the realization flaws.

An SA model has properties such as performance or security. Regularly, these properties are emergent, and it is more feasible to reason about emergent properties in simpler models than complex ones. So, it is needed to simplify your model to leverage the problem and prove your knowledge about the emergent properties [107].

## 6. Identified Features and Factors

**Figure 3:**
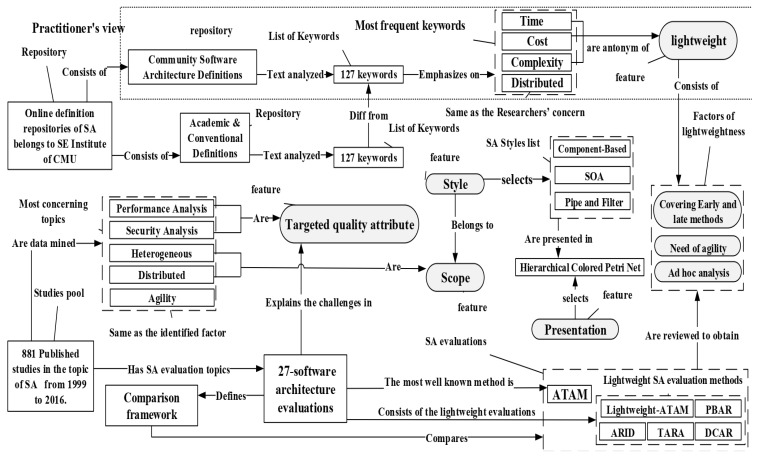
Identifying features and lightweight factors.

The study was devised to identify the features and lightweight factors to boost an evaluation framework. Concerning this fact, the study sought the practitioner’s needs and the tendencies that researchers have paid attention to. Figure 3 shows the relationships and basis for identifying the features and factors. Then Table 6 lists the specified features and lightweight characteristics acquired from the study. Figure 3 shows how this research applies text analysis and data mining to the comprehensive online definitions of SA and the pool of papers published in the last three decades. In the next step, a comparison framework was defined, and six approaches were inspected deeply to find features and factors.

First of all, two categories of SA definition were elicited from the online repository. In the next step, the keywords in practitioners’ reports differed from the keywords of researchers. The top keywords are “time, cost, distributed, and complexity”, which means the practitioners needed a lightweight solution. “Distributed” refers to the scope of the evaluation. Secondly, based on the 811 published studies from 1999 to 2016 in SA’s topics, “security and performance analysis, heterogeneity and distributed, and agility” were the most popular research topics. Similar to a practitioner’s concerns, heterogeneity and distribution refer to the scope. Security and performance were selected as the targeted quality attributes (TQA) for evaluation. Agility was the same as one of the identified factors for lightweights.

Component-Based architecture, SOA, and pipe and filter styles were selected as the identified scope’s proper SA styles. As mentioned in Section 3, Section 4 and Section 5.1, Petri net’s formalism and visual presentation, hierarchical colored Petri net, is chosen to present SA due to its supremacy over other SA presentation methods.

For the sake of research concerns, 76 articles with SA evaluation topics were selected out of 811 articles. Next, 27 SA evaluation methods were chosen for the review. These methods were reviewed based on the following two aspects: the technique and popularity in the industry. The comparison framework was defined to compare the evaluation methods. Then five lightweight methods were selected and compared with ATAM. Consequently, three lightweight factors were identified. Moreover, (TQA1) performance and (TQA2) security were reviewed throughout 27 evaluation methods. As mentioned many times in this study, the SA evaluation approaches are devised to help software architects make a proper decision. Obviously, the architects are interfering in many steps to heighten the evaluation process, so the SA evaluation tends to be more manual rather than automated. Architects’ skills may impact the design and decision-making parts that are outside the scope of this research.

Based on identified features, factors, and comparison framework, the overall profile of a lightweight evaluation framework is described below as follows:

This study replied to research question two by distinguishing the differences between practitioners’ and researchers’ perspectives on SA via the comparative text analysis of SA definitions and the systematic literature review of existing methods. Then, for the sake of the first research question, this exploratory research identified the features and characteristics that enable lightweight SA evaluations in the industry. An evaluation framework can boost its usage by detecting flaws and issues in SA’s performance and security. An informal description of requirements, UML diagrams, and source code is the input of the framework. The framework works within the specific scope of distributed software with the mentioned SA styles. The procedure for stakeholders is the minimal process, which was elicited from reviewing the lightweight solutions. The procedure is a face-to-face meeting between the architect and internal/external reviewers who know SA. The tools and techniques should be investigated to ease the integration of features and factors.

## 7. Achievement and Results

The existing literature was reviewed to remind us of the past research on using SA evaluation methods to identify the proposed framework’s main features in the first stage. In the second stage, the factors affecting lightweights are identified. These factors improve the SA evaluation framework used in the industry.

In the first stage, the SA evaluation framework’s features were identified based on the text analysis of researchers’ and practitioners’ SA definitions and all published studies for the last three decades on SA’s topics. This analysis concluded that a lightweight SA evaluation solution was needed to uncover distributed and heterogeneous software’s security and performance problems. Consequently, the security and performance analysis of SA were reviewed, and the proper SA presentation and styles for the distributed and heterogeneous software were identified.

In the second stage, lightweights were identified from the weaknesses of the current state of the art in the lightweight SA analysis methods. Indeed, the study tried to bridge the gap of less usage of systematic SA evaluations in the industry. First, it should be clear why the industry refrains from SA evaluation methods proposed by academics. As a result, this study followed two strands of academic and practitioner concerns. The practitioners need the SA evaluation framework with specific industrial features to solve their current problems, while academics focus primarily on scientific issues and possible issues in the future. This mindset led us to analyze the online web repository of SA definitions. As a result of that, the main extracted features that are in demand for both sides were a lightweight framework that can evaluate heterogeneous software systems from a performance and security perspective.

Moreover, the study conducted a systematic literature review on SA evaluation methods. As a result, the SA evaluation comparison framework was proposed as a basis for the SA evolution comparison. Then, it narrowed down the literature to the lightweight SA evaluation methods. A total of six SA evaluation methods were studied deeply to identify the factors influencing the SA evaluation method.

## 8. Conclusions

Although SA evaluation methods are beneficial, they are not broadly applied in the industry [108,109]. The selected SA evaluation methods are reviewed comprehensively. This research focuses on SA architecture, design, and its evaluation. It introduces the comparison framework to compare existing methods. The comparison between ATAM, as the heavyweight method’s pinnacle, and the five fashionable lightweight methods recognizes three main factors for lightweights. A total of five different steps have been taken to address this problem. Firstly, the differences between academic and practitioner definitions of SA prove that the industry needs a lightweight SA evaluation method. Secondly, it is noticed that SA research mainly focuses on “performance and security analysis”. Finally, these are the main features and factor that have been identified. As a result, the literature review explored SA evaluation methods to categorize them to understand the factors that hinder the lightweight SA evaluation method’s success. The research suggests further investigation to find the proper tools and techniques to ease the integration of features and factors and boost solution usage in the industry.

## Figures and Tables

**Table 1:** The selected published papers in SA evaluation.

Reference Study	Focus
Breivold et al. [49]	The search identified 58 studies that were cataloged as primary studies for this review after using a multi-step selection process. The studies are classified into the following five main categories: techniques supporting quality considerations during SA design, architectural quality evaluation, economic valuation, architectural knowledge management, and modeling techniques.
Barcelos et al. [50]	A total of 11 evaluation methods based on measuring techniques are used, mainly focusing on simulation and metrics to analyze the architecture.
Suman et al. [51]	This paper presents a comparative analysis of eight scenario-based SA evaluation methods using a taxonomy.
Shanmugapriya et al. [52]	It compares 14 scenario-based evaluation methods and five of the latest SA evaluation methods.
Roy et al. [53]	The taxonomy is used to distinguish architectural evaluations based on the artifacts on which the methods are applied and two phases of the software life cycle.
Mattsson et al. [54]	The paper compares 11 various evaluation methods from technical, quality attributes, and usage views.
Hansen et al. [55]	The research reports three studies of architectural prototyping in practice, ethnographic research, and a focus group on architectural prototyping. It involves architects from four companies and a survey study of 20 practicing software architects and developers.
Gorton et al. [56]	This paper compares four well-known scenario-based SA evaluation methods. It uses an evaluation framework that considers each method for context, stakeholders, structure, and reliability.
Weiss et al. [57]	It conducted a survey based on architectural experience, which was organized into six categories. The architecture reviews found more than 1000 issues between the years 1989 and 2000.
Babar et al. [58]	It discusses the agility in SA evaluation methods.
Suryanarayana et al. [59]	It states refractory adaption for architecture evaluation methods.
Lindvall et al. [60]; Santos et al. [61]	These are references of reviewed papers lacking stated knowledge, which is needed in the paper or more investigation.
Oliveira et al. [62]	It reviews the agile SA evaluation.
Martensson et al. [63]	It reviews the scenario-based SA evaluation based on industrial cases.

**Table 3:** Software evaluations comparison framework.

Comparison Framework			
Component	Elements	Brief Explanation	Taxonomic Comparison
Context	SA definition	Does the method overtly consider a specific definition of SA?	NA
	Specific goal	What is the specific aim of the methods?	Need for Evolution: Corrective, Perfective, Adaptive, Preventive, All applicable.
			Means of Evaluation (Static): Transformation, Refactoring, Refinement, Restructuring, Pattern change.
			Means of Evaluation (Dynamic): Reconfiguration, Adaptation
	Quality attributes	How many and which QAs are covered by the method?	QAs
	Applicable stage	Which is the most suitable development phase for applying the method?	Early
			Late
	Input and output	What are the required inputs and produced outputs?	In: coarse-grained, medium, or fine SA design, views
			Out: risks, issues
	Application domain	What is/are the application domain(s) the method is often applied?	Development Paradigm: SPL, OO, SOA, CBS
			Traditional: Embedded, Real-time, Process-aware, Distributed, Event-based, Concurrent, Mechatronic, Mobile, Robotic, Grid computing
			Emerging: Cloud computing, Smart grid, Autonomic computing, Critical system, Ubiquitous
	Benefits	What are the advantages of the method to the stakeholders?	NA
Stakeholder	Involved Stakeholders	Which groups of stakeholders are needed to take part in the evaluation?	NA
	Process support	How much support is supplied by the method to perform various activities?	NA
	Socio-technical issues	How does the method handle non-technical (e.g., social, organizational) issues?	NA
	Required resources	How many man-days are needed? What is the size of the evaluation team?	NA
Contents	Method’s activities	What are the activities to be accomplished, and in which order to reach the aims?	Means of Evolution: Static, Dynamic
			Support activity: Change impact, Change history
	SA description	What form of SA description is needed (e.g., formal, informal, ADL, views, etc.)?	Type of Formalism
			Description of Language
			UML Specification
			Description Aspect
	Evaluation approaches	What are the types of evaluation approaches applied by the method?	Experience-based, Prototyping-based, Scenario-based, Checklist-based, Simulation-based, Metrics-based, Math Model-based
	Tool support	Are there tools or experience repositories for supporting the method and its artifacts?	Need for Tool Support Analysis
			Usage of Tool Support
			Level of Automation
Reliability	Maturity of the method	What is the level of maturity (inception, refinement, development, or dormant)?	Overview or Survey, Formalism for constraint specification, Formalism for architectural analysis, Formalism for arch and evolution, Formalism for code generation.
	Method’s validation	Has the method been validated? How has the method been validated?	Case study, Mathematical proof, an Example application, Industrial validation
Time	Time of evaluation	Stage of evolution	Early, middle, and Post-deployment.
		SLDC	Analysis/Design, Implementation, Integration/provisioning, Deployment, Evolution
		Specification-time	Design-Time, Run-Time
**Complementary table**			
Means of Evolution:		Static: Transformation, Refactoring, Refinement, Restructuring, Pattern change	
		Dynamic: Reconfiguration, Adaptation	
Support activity:		Change impact: Consistency checking, Impact analysis, Propagation;	
		Change history: Evolution analysis, Versioning	
Type of Formalism		Modeling language: ADL, Programming languages, Domain-specific language, Type systems, Archface, Model-based	
		Formal models: Graph theory, Petri-net, Ontology, State machine, Constraint automata, CHAM	
		Process algebra: FSP, CSP, π-calculus; Logic (Constraint language): OCL, CCL, FOL, Grammars, Temporal logics, Rules, Description logic, Z, Alloy, Larch	
Description of Language		Process algebra: Darwin, Wright, LEDA, PiLar	
		Standards: UML, Ex.-UML, SysML, AADL	
		Others: ACME, Aesop, C2, MetaH, Rapide, SADL, UniCon, Weaves, Koala, xADL, ADML, AO-ADL, xAcme	
UML Specification		Static: Class, Component, Object.	
		Dynamic: Activity, State, Sequence, Transition, Communication	
Description Aspect		Structural, Behavioral, Semantic	
Need for Tool Support Analysis		Architecture lifecycle: Business case, Creating architecture, Documenting, Analyzing, Evolving	
Usage of Tool Support		Simulation, Dependence analysis, Model checking, Conformance testing, Interface consistency, Inspection, and Review-based	
Automation’s level		Fully automated, Partially automated, Manual	

**Table 5:** Lightweight methods comparison.

Aspect	Category	Approaches
The goal of the evaluation method	Assessment against requirements	Lightweight ATAM
	Architectural flaws detection	PBAR, TARA, ARID
SA description	Architectural decisions	DCAR
	Full SA description (views)	Lightweight-ATAM
	SA patterns, tactics	PBAR
	There is no specific form of SA designs or documents	ARID, PBAR, TARA
Time of evaluation	Early	DCAR
	Middle	ARID, PBAR, Lightweight-ATAM
	Post-deployment	TARA
Method’s validation based on case study number	0	Lightweight-ATAM
	1	TARA
	3 to 6	PBAR, DCAR
	6+	ATAM
Tool Support	Conformance testing	TARA
	Review-based	DCAR

**Table 6:** The identified features and lightweight’s factors.

Features		Lightweights Factors
The excess of the SA Evaluation work:	Lightweight	Covering Early and late methods
		Need of agility
		Ad hoc analysis
Scope of the SA Evaluation:	Distributed and heterogamous system	
Style of evaluated SA:	SOA, Component-based pipe and filter	
SA presentation:	Petri nets	
Targeted quality attributes:	Performance and security	
Tools and technique:	Should be investigated	

## Data Availability

Not applicable.

## Abbreviations

**Symbol**	**Meaning**
AABI	Andolfl et al.
ABAS	Attribute based Architectural Style
ABI	Aquilani et al.
ACP	Algebra of Communicating Processes
ADL	Architecture Description Language
AELB	Atomic Energy Licensing Board
ARCADE	Architecture Recovery, Change, and Decay Evaluator
ARID	Active Reviews for Intermediate Designs
ATAM	Architecture-based Tradeoff Analysis Method
BIM	Balsamo et al.
CCS	Calculus of Communicating Systems
CM	Cortellessa and Mirandola
CMMI	Capability Maturity Model Integration
CPN	Colored Petri nets
CSP	Communicating Sequential Processes
DCAR	Decision-Centric Architecture Reviews
EBAE	Empirically Based Architecture Evaluation
FERA	Framework of evaluation of Reference Architectures
GQM	Goal Question Metrics Paradigm
GUI	Graphical User Interface
HCPN	Hierarchical Colored Petri nets
HM	Heuristic Miner
LQN	Layered Queuing Networks
LTL	Linear Temporal Logic
LTS	Labeled Transition System
MVC	Model View Controller
OWL	Web Ontology Language
PAIS	Process Aware Information System
PASA	Performance Assessment of Software Architecture
PBAR	Pattern-Based Architecture Reviews
PM	Process Mining
QNM	Layered Queuing Networks
RARE	Reference Architecture Representation Environment
REST API	Representational State Transfer Application Programming Interface
SA	Software Architecture
SAAM	Scenario-based Software Architecture Analysis Method
SACMM	Metrics of Software Architecture Changes based on Structural Metrics
SADL	Simulation Architecture Description Language
SAM	Formal systematic software architecture specification and analysis methodology
SAR	Software Review Architecture
SOA	Service Oriented Architecture
SPE	Software Performance Analysis
TARA	Tiny Architectural Review Approach
UML	Unified Modeling Language
WS	Williams and Smith
SPL	Software Product Line
OO	Object Oriented
CBS	component based Software
